# Novel candidate genes for vestibular function identified through GWAS in the hybrid mouse diversity panel

**DOI:** 10.1186/s12864-026-12810-y

**Published:** 2026-04-11

**Authors:** Yuzuru Ninoyu, Calvin Pan, Jennifer Luu, Sameeha Rashid, Jadyn Johnston, Briana Ortega, Ely Boussaty, Lu Lu, Royce Clifford, Pejman Mohammadi, Francesca Telese, Abraham A. Palmer, Aldons J. Lusis, Rick A. Friedman

**Affiliations:** 1 Department of Otolaryngology - Head and Neck Surgery, University of California San Diego, La Jolla, CA USA; 2 Department of Otolaryngology - Head and Neck Surgery, Kyoto Prefectural University of Medicine, Kyoto, Japan; 3 Department of Medicine, Division of Cardiology, David Geffen School of Medicine, University of California, Los Angeles, Los Angeles, CA USA; 4 Department of Anatomy and Neurobiology, University of Tennessee Health Science Center, Memphis, TN USA; 5 Center for Immunity and Immunotherapies, Seattle Children’s Research Institute, Seattle, WA USA; 6 Department of Pediatrics, University of Washington School of Medicine, Seattle, WA USA; 7 Department of Psychiatry, University of California San Diego, La Jolla, CA USA; 8 Institute for Genomic Medicine, University of California San Diego, La Jolla, CA USA

**Keywords:** vestibular function, Genome-wide association studies (GWAS), Cochlea

## Abstract

Disequilibrium is a highly prevalent age-related condition that increases fall risk, yet the genetic architecture underlying vestibular function remains poorly defined. Here, we performed genome-wide association studies (GWAS) of vestibular-evoked potentials (VsEP) and raised-beam performance across young and aged Hybrid Mouse Diversity Panel (HMDP) strains. In young mice, we identified genome-wide significant loci on chromosomes 4, 7, 14, and 15, along with suggestive associations on chromosomes 6 and 14. To refine candidate genes, we integrated cochlear and cerebellar cis-eQTL data from BXD strains with human cochlear transcriptomic profiles, revealing 12 cochlea-enriched genes within linkage disequilibrium intervals. Single-cell RNA sequencing localized *Agbl4*, *Cntnap2*, *Dmc1*, and *Pcdh20* to vestibular hair cells, and *Gpnmb* to melanocytes of the inner ear. These findings highlight coordinated contributions of sensory and non-sensory cell types to vestibular performance. Although no significant loci were detected in aged mice, our integrative genetic and transcriptomic framework provides new insight into the molecular architecture of vestibular function and potential pathways underlying age-related balance impairment.

**Supplementary Information:**

The online version contains supplementary material available at 10.1186/s12864-026-12810-y.

## Background

Disequilibrium, defined as a non-vertiginous disturbance of static or dynamic postural balance, is a common age-related condition. It affects roughly 30% of adults over 60 years of age and nearly half of those older than 85 years [1, 2]. It greatly reduces quality of life and increases the risk of falls, which are a leading cause of morbidity in the elderly [3, 4]. The causes of disequilibrium are multifactorial, including declines in muscle strength, motor coordination, and vestibular function. In particular, age-related dysfunction of the otolithic organs—the gravity receptors of the vestibular system—has been closely linked to falls, with evidence of degeneration in vestibular hair cells (HCs), otoconia, afferent fibers, and Scarpa’s ganglion neurons [5, 6].

Despite its clinical importance, the genetic basis of vestibular dysfunction and its age-related progression remains poorly understood. Genome-wide association studies (GWAS) have implicated polygenic factors in conditions such as motion sickness and vestibular neuronitis, but progress in identifying genetic contributors to vestibular decline has been limited [7–10]. These studies collectively suggest that vestibular-related phenotypes have a heritable and polygenic component, although direct physiological measures of vestibular function have rarely been investigated. Identifying the genetic factors underlying vestibular decline could enable earlier prediction of susceptibility, support personalized risk assessment, and reveal novel molecular targets for therapeutic intervention.

To address this gap, we utilized the Hybrid Mouse Diversity Panel (HMDP), comprising 84 inbred strains of young mice and 94 of aged mice, to investigate the genetic determinants of vestibular function. Because human vestibular GWAS have been limited by phenotypic variability and difficulties in precisely quantifying vestibular performance, the HMDP provides a powerful alternative model with controlled genetic backgrounds and reproducible phenotyping [11–13].

Importantly, the use of genetically distinct inbred strains enables estimation of heritability and facilitates mapping of naturally segregating alleles contributing to quantitative variation in vestibular traits. Vestibular performance was evaluated using vestibular evoked potentials (VsEPs), which measure otolith (utricle and saccule) function, and the balance beam test, which reflects integrated vestibular, visual and motor performance. These traits were then used for GWAS to identify associated loci. To prioritize and interpret candidate genes, we integrated publicly available gene expression data from cochlear and cerebellar tissues in the HMDP and identified genes with significant cis-eQTLs. We further refined these candidates by overlapping them with human cochlear gene expression profiles [14]. Finally, we examined the expression patterns of prioritized genes using publicly available single-cell RNA sequencing (scRNA-seq) datasets from cochlear and vestibular tissues. This integrated genetic and transcriptomic approach enabled the identification of novel candidate genes underlying vestibular function.

## Results

### Phenotypic diversity of VsEP and balance beam test among HMDP strains

**Fig. 1:**
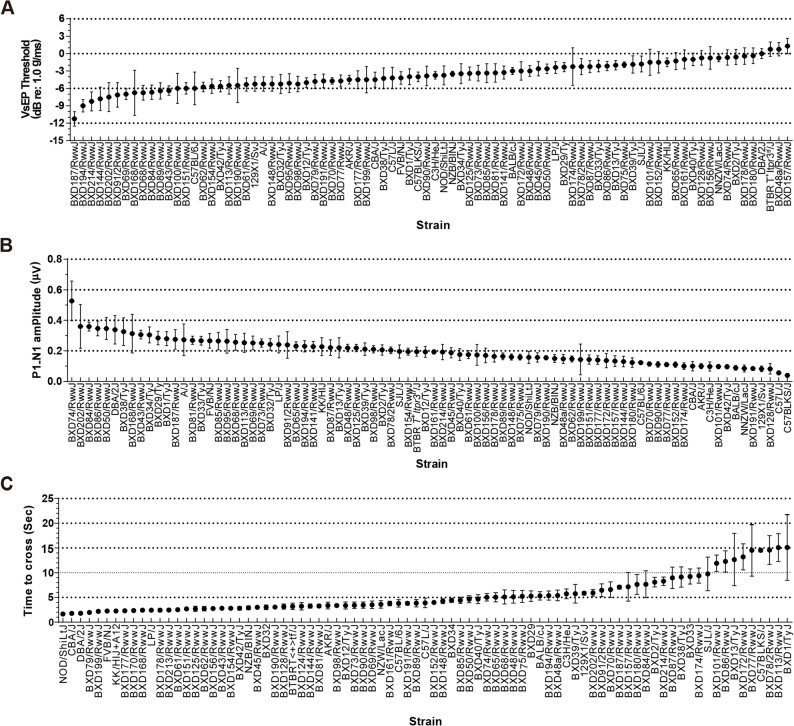
Phenotypic diversity of vestibular and balance performance among HMDP inbred mouse strains. **A–B** Vestibular sensory function was assessed by vestibular sensory evoked potentials (VsEPs) in 6-week-old female mice from 84 Hybrid Mouse Diversity Panel (HMDP) strains (*n* = 544; 5–7 mice per strain; see Supplemental Table 1 for detailed strain information, sample size, and values). Parameters included (A) VsEP threshold (dB re: 1.0 g/ms) and (**B**) P1–N1 amplitude (µV) in 6-week-old female HMDP mice. A broad range of VsEP thresholds was observed (mean ± SEM: − 3.79 ± 3.36 dB), varying from − 11.3 ± 2.49 dB in BXD187/RwwJ to 1.29 ± 3.53 dB in BXD157/RwwJ, indicating substantial genetic influences on gravity-receptor sensitivity. The mean P1–N1 amplitude was 0.20 ± 0.085 µV, spanning over a four-fold range, with BXD74/RwwJ showing the largest amplitude (0.53 ± 0.39 µV) and C57BLK/J (0.04 ± 0.022 µV) and C57L/J (0.06 ± 0.030 µV) displaying minimal responses. **C** Vestibulo-motor performance was evaluated using the raised-beam balance test (*n* = 444) in 6-week-old female HMDP mice\. Traversal times varied markedly across strains, from 1.65 ± 0.11 s in NOD/ShiLtJ and 1.77 ± 0.25 s in CBA/J to over 15 s in BXD1/TyJ (15.1 ± 13.3 s) and BXD113/RwwJ (15.1 ± 6.3 s). Detailed strain-level data are provided in Supplemental Table 2

Vestibular function in young mice was evaluated in 6-week-old female mice from 84 HMDP strains (*n* = 504) using VsEP thresholds and P1–N1 amplitude (Supplemental Table 1), while vestibulo-motor performance was assessed from 83 strains (*n* = 413) using the raised-beam assay (Supplemental Table 2). A broad distribution of VsEP thresholds was observed across strains (mean − 3.79 ± 3.36 dB re: 1.0 g/ms) (Fig. 1A), indicating substantial genetic influences on gravity receptor sensitivity. The mean P1–N1 amplitude was 0.20 ± 0.085 µV (Fig. 1B), spanning more than a fourfold range. Performance on the balance beam test reflected similar diversity (Fig. 1C). Together, these findings demonstrate substantial strain-dependent variation in vestibular sensitivity and vestibulo-motor coordination in young mice. Broad-sense heritability estimates were H^2^ = 0.20 for VsEP threshold, H^2^ = 0.25 for P1-N1 amplitude, and H^2^ = 0.46 for beam performance, confirming a measurable genetic contribution and providing sufficient heritable variation for downstream GWAS.

**Fig. 2:**
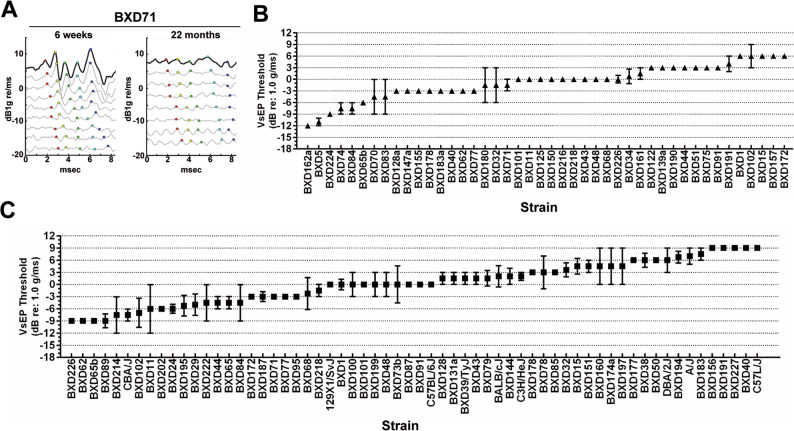
Age-related and strain-dependent differences in vestibular sensory evoked potentials (VsEPs) among HMDP strains. **A** Representative VsEP waveforms recorded from 6-week-old (young) and 22-month-old (aged) BXD77 mice (female). Each dot marks a wave peak (I–V), identified using standard VsEP waveform analysis procedures (see Methods). Compared with the young mouse, the aged mouse exhibited elevated thresholds and reduced amplitudes, illustrating typical age-related decline in vestibular sensitivity. **B**,** C** VsEP thresholds (dB re: 1.0 g/ms) were compared among aged male **(B)** and aged female **(C)** mice from the Hybrid Mouse Diversity Panel (HMDP) (*n* = 44 and 62 strains, respectively). Data represent mean ± SEM for each strain; in some strains, only a single mouse was available. Exact strain names, sample sizes, and threshold values are provided in Supplemental Table 3

To investigate genetic determinants of vestibular function in aging, VsEP thresholds were measured separately in aged female and male mice, representing 62 and 44 strains, respectively (Supplemental Table 3). Because strain availability differed between sexes due to strain-dependent survival, the two groups were analyzed independently. Aged mice exhibited the expected age-related decline in vestibular sensitivity, with elevated thresholds and reduced amplitudes (Fig. 2A). Despite this overall decline, aged mice showed substantial strain-dependent variability, with the mean thresholds of 0.41 dB re: 1.0 g/ms in females and 0.76 dB re: 1.0 g/ms in males (Fig. 2B). One-way ANOVA demonstrated significant effects of strain in both sexes (females: F_61,83_ = 2.86, *p* = 5 × 10^− 6^; males: F_43,23_ = 2.88, *p* = 0.004), confirming robust genetic contributions to aged vestibular sensitivity. Broad-sense heritability estimates were similarly high (H^2^ = 0.44 in females; H^2^ = 0.51 in males), indicating that genetic background explains a substantial proportion of phenotypic variance in aged VsEP thresholds. Thus, even late in life, vestibular function remains strongly shaped by genetic factors.

### GWAS for VsEP and raised-beam test identified significant peak SNPs

We performed GWAS for VsEP thresholds, P1-N1 amplitude, and raised-beam traverse time using FaST-LMM. The young cohort consisted exclusively of female mice, whereas aged males and females were analyzed separately. In the aged cohort, the strain composition differed between sexes due to strain-dependent survival and availability; therefore, male and female GWAS were conducted as independent analyses rather than direct sex comparisons. Following standard SNP filtering (MAF of ≥ 5%; missing rate ≤ 10%), ~ 104,000-166,000 SNPs were analyzed depending on strain composition (Supplemental Table 4). Genome-wide and suggestive significance thresholds were set at *p* = 4.1 × 10^− 6^ (-log_10_(*p*) = 5.39) [13], and *p* ≤ 1.0 × 10⁻⁵, respectively.

Genomic inflation factors (λGC) varied across traits (Supplemental Fig. 1). VsEP thresholds in young mice showed minimal inflation (λGC = 1.012), whereas P1-N1 amplitude (λGC = 1.360), raised-beam performance (λGC = 1.251), and aged-male thresholds (λGC = 1.310) exhibited higher inflation levels. Aged-female thresholds showed modest inflation (λGC = 1.113). In the HMDP, moderate λGC inflation is typically interpreted as reflecting true polygenic signal rather than uncontrolled population structure, because FaST-LMM accounts for relatedness through a kinship matrix. Consistent with this, traits with higher λGC values also showed stronger association peaks, supporting the presence of polygenic architecture rather than confounding effects.

**Fig. 3:**
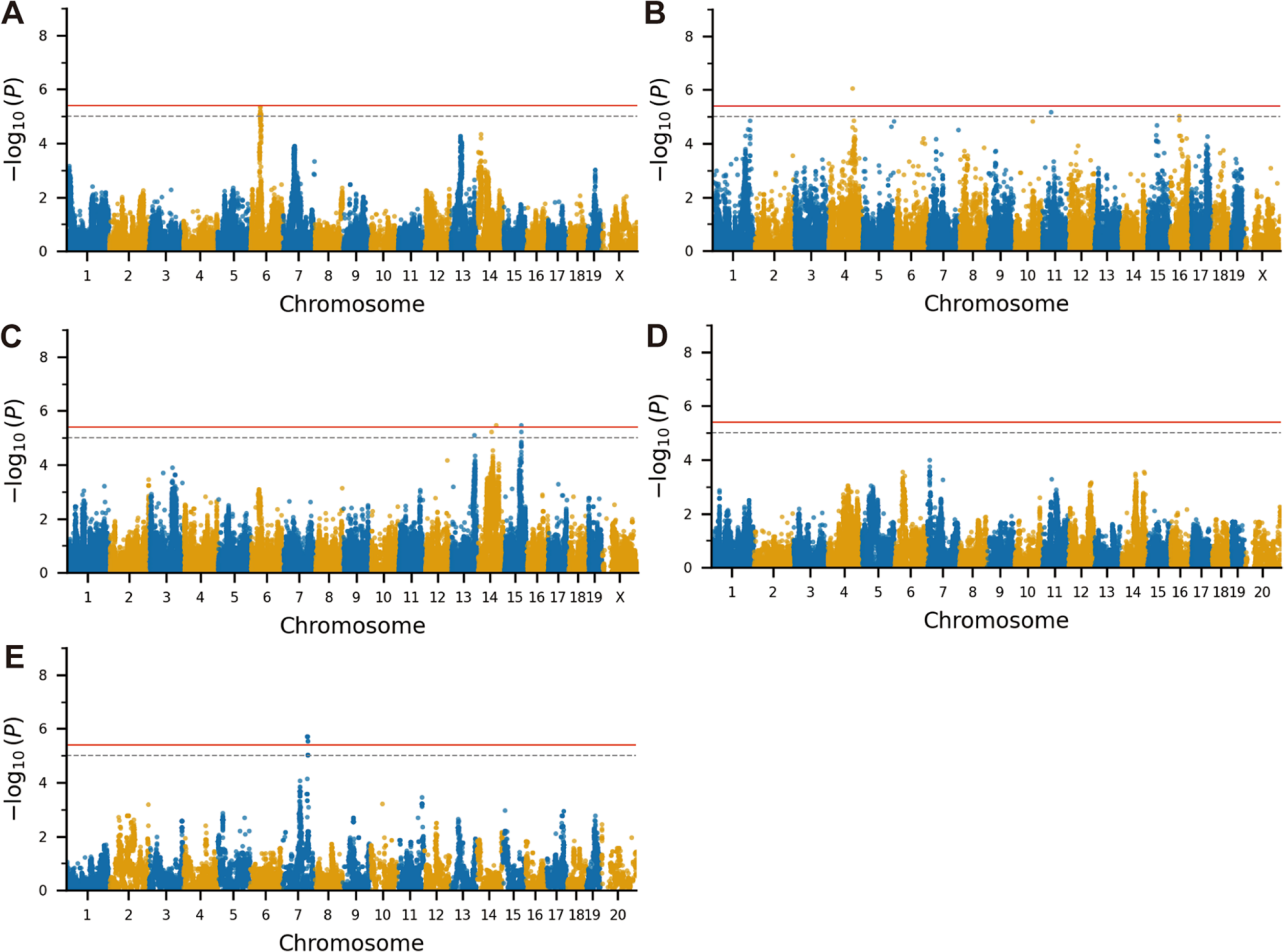
Genome-wide association analyses of vestibular and balance phenotypes in HMDP mice. **A–E** Manhattan plots of genome-wide association studies (GWAS) for vestibular and balance traits in the Hybrid Mouse Diversity Panel (HMDP). Each point represents a single nucleotide polymorphism (SNP) plotted by chromosomal position (x-axis) and –log₁₀ *p* value (y-axis). The red horizontal line indicates the genome-wide significance threshold (–log₁₀ *p* = 5.39), and the black dashed line denotes the suggestive significance level (–log₁₀ *p* = 5.00). **(A)** VsEP threshold, **(B)** P1–N1 amplitude, and **(C)** balance-beam traversal time in young (6-week-old) mice showed association peaks on chromosomes 6, 4, 14, and 15, respectively. The chr6 locus exhibited a suggestive association (–log₁₀ *p* = 5.35), while significant peaks were detected on chr 4 (–log₁₀ *p* = 6.06), chr 14 (–log₁₀ *p* = 5.47), and chr 15 (–log₁₀ *p* = 5.47). **D**,** E** VsEP threshold GWAS in aged cohorts showed no genome-wide significant peaks in aged females **(D**), whereas aged males **(E)** displayed a significant association on chr 7 (–log₁₀ *p* = 5.47). The exact SNP identifiers, chromosomal positions, and -log_10_ *p* values for each lead locus are listed in Table 1.

Several significant and suggestive association peaks were identified across multiple chromosomes (Fig. 3; Table 1.). Significant associations were detected for P1-N1 amplitude on Chr. 4 (rs28252548; -log_10_(*p*) = 6.060) (Fig. 3B), for raised-beam traverse time on Chr. 14 (rs30866996; -log_10_(*p*) = 5.47) and Chr. 15 (rs31521941; -log_10_(*p*) = 5.47) (Fig. 3C), and for aged-male VsEP thresholds on Chr. 7 (rs31152067; -log_10_(*p*) = 5.72) (Fig. 3E). Suggestive associations were identified on Chr. 6 for young VsEP thresholds (rs30360878 and rs51387934; -log_10_(*p*) = 5.35) (Fig. 3A) and on Chr. 14 for raised-beam performance (rs30397233; -log_10_(*p*) = 5.21) (Fig. 3C).

**Table 1.** Lead SNPs and genomic loci identified by GWAS of vestibular and balance traits in HMDP mice

Lead SNP	SNP location(Mb)	-log(p)	Genomic context	Trait
rs28252548	Chr4 : 111.486380	**6.06**	Intronic (Agbl4)	VsEP P1-N1 amplitude
rs51387934	Chr6 : 47.190920	5.35	Intronic(Cntnap2)	VsEP threshold, young
rs30360878	Chr6 : 48.206698	5.35	Intergenic	VsEP threshold, young
rs31152067	Chr7 : 115.799898	**5.72**	Intronic (Sox6)	VsEP threshold, aged, male
rs30397233	Chr14: 68.700488	5.21	Intergenic	Raised-beam test
rs30866996	Chr14: 89.667363	**5.47**	Intergenic	Raised-beam test
rs31521941	Chr15: 78.234670	**5.47**	Intergenic	Raised-beam test

**Fig. 4:**
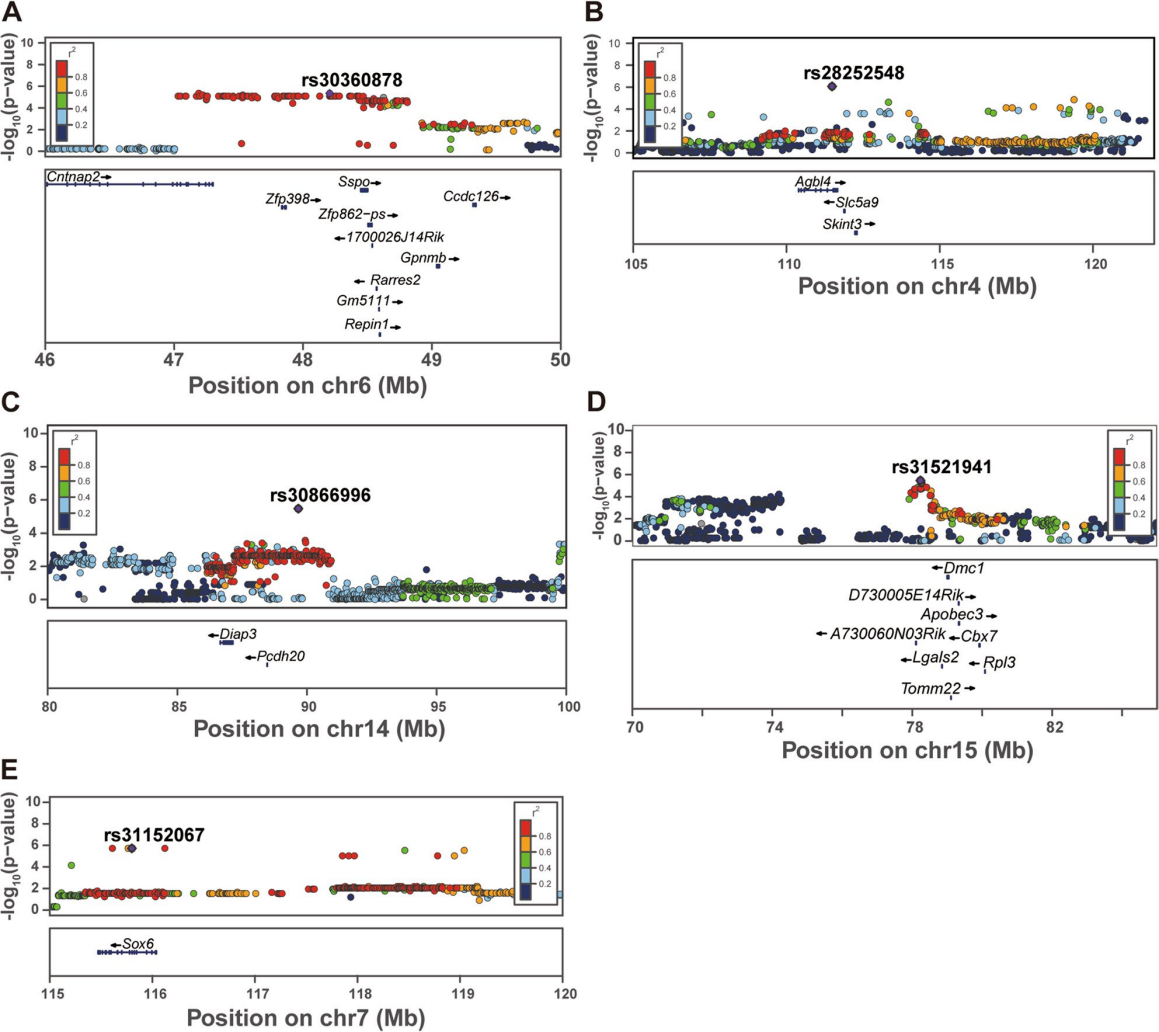
LocusZoom plots of genomic regions associated with vestibular and balance phenotypes in HMDP mice. **A–E** Regional association plots showing linkage disequilibrium (LD) structure surrounding the lead SNPs identified in genome-wide association analyses. Each point represents a single nucleotide polymorphism (SNP), plotted by chromosomal position (x-axis) and –log₁₀ P value (y-axis). SNPs are color-coded according to their LD (r²) with the lead SNP (shown in purple). **(A)** Chr 6 locus (lead SNP rs30360878) from the VsEP threshold GWAS, **(B)** Chr 4 locus (rs2825248) from the P1–N1 amplitude GWAS, **(C)** Chr 14 locus (rs30866996) and **(D)** Chr 15 locus (rs31521941) from the balance-beam performance GWAS, and **(E)** Chr 7 locus (rs31152067) from the aged male VsEP threshold GWAS. Genes located within regions of LD, including those showing cis-eQTL associations, are indicated. The well-known hereditary hearing loss gene *Diap3* is also highlighted within the Chr 14 locus despite the absence of a cis-eQTL signal

Visualization of regional association patterns using Locus Zoom illustrated distinct linkage disequilibrium (LD) structures surrounding each lead SNP, delineating candidate genomic intervals for downstream gene prioritization (Fig. 4). For P1-N1 amplitude, the significant association on Chr. 4 was located within an intron of *Agbl4*, a gene enriched in the postnatal utricular HC [15] (Fig. 4B). For raised-beam traverse time, the Chr. 14 peak encompassed *Diap3*, a gene previously linked to progressive hearing loss [16], and *Pcdh20*, which has been reported in human GWAS as a susceptibility gene for hearing function [17] (Fig. 4C). In the VsEP threshold GWAS of aged mice, no significant peak was found in females, whereas a significant association was detected on Chr. 7, located within *Sox6*, in males (Fig. 4E).

### Integration of GWAS and cis-eQTL analyses

To prioritize candidate genes within the loci identified by the GWASs, we examined publicly available gene expression data from BXD mouse strains in the cochlea and cerebellum using the GeneNetwork database (https://genenetwork.org/). Genes located within the LD block of the lead SNPs (r² > 0.8) were evaluated for expression quantitative trait loci (eQTLs). A locus was classified as cis-acting when the eQTL was located within ± 2 Mb of the peak GWAS SNP and had a q-value < 0.05 (− log₁₀(*q*) > 1.30). Several GWAS loci demonstrated significant cis-eQTLs in the cochlear tissue (Table 2). For instance, *Agbl4* (Chr. 4, rs28303726; −log₁₀(*q*) = 3.11) showed a strong cis-eQTL corresponding to the P1–N1 amplitude GWAS. Similarly, *Gpnmb* (Chr. 6, rs30015053; −log₁₀(*q*) = 10.96) was associated with VsEP threshold in young mice, and *Adam28* (Chr. 14, rs3089069; −log₁₀(*q*) = 3.28) was linked to beam test performance. Although *Cntnap2* did not display a cochlear cis-eQTL, it exhibited a significant eQTL in the cerebellum (rs45093870; −log₁₀(*q*) = 7.89). In the aged-male VsEP threshold GWAS, a significant intronic variant within *Sox6* was identified; although *Sox6* itself lacked cis-eQTLs in both tissues, nearby genes (*Cyp2r1* and *Spon1*) showed significant cis-eQTLs in both the cochlea and cerebellum. We identified a total of 23 candidates of cis-eQTL genes.

**Table 2:** cis-eQTL genes located within ±2 Mb of lead SNPs identified in vestibular GWAS loci

**Lead SNP**	**MAF**	**SNP location**	**eQTL Lead SNP**	**MAF**	**eQTL location**	**Candidate gene**	**Gene location**	**eQTL q-value (-log10(q))**	**eQTL tissue**
rs28252548	0.39	Chr4 : 111.486380	rs28303726	0.36	Chr4 : 111.311031	*Agbl4*	Chr4 : 110.397661	3.11	Cochlea
						*Skint3*	Chr4 : 112.232245	8.45	Cochlea
						*Slc5a9*	Chr4 : 111.875375	8.45	Cochlea
rs30360878	0.5	Chr6 : 48.206698	rs30074265	0.48	Chr6 : 47.102308	*Cntnap2*	Chr6 : 45.059357	7.89	Cerebellum
						*Zfp398*	Chr6 : 47.835661	2.94	Cochlea
								10.73	Cerebellum
			rs37440219	0.43	Chr6 : 48.436632	*Sspo*	Chr6 : 48.448229	2.44	Cochlea
						*1700026J14Rik*	Chr6 : 48.536582	5.40	Cochlea
						*Rarres2*	Chr6 : 48.569696	5.10	Cochlea
						*Gm5111*	Chr6 : 48.589445	2.69	Cochlea
			rs13478739	0.48	Chr6 : 47.027244	*Gpnmb*	Chr6 : 49.036546	10.96	Cochlea
			rs30015053	0.47	Chr6 : 48.921901	*Ccdc126*	Chr6 : 49.319274	3.62	Cochlea
rs31152067	0.36	Chr7 : 115.799898	rs32471282	0.48	Chr7 : 114.616126	*Cyp2r1*	Chr7 : 114.550201	6.17	Cochlea
						*Spon1*	Chr7 : 114.042011	4.13	Cerebellum
rs30866996	0.45	Chr14 : 89.667363	rs31432790	0.43	Chr14 : 89.749066	* Pcdh20*	Chr14 : 88.466589	2.19	Cerebellum
rs30397233	0.47	Chr14 : 68.700488	rs3089069	0.47	Chr14 : 68.761776	*Adam28*	Chr14 : 68.606027	3.28	Cochlea
rs31521941	0.4	Chr15 : 78.234670	rs6342608	0.37	Chr15 : 78.159752	*A730060N03Rik*	Chr15 : 78.119706	2.58	Cochlea
						*Lgals2*	Chr15 : 78.85086	3.97	Cochlea
						*Apobec3*	Chr15 : 79.286228	7.69	Cochlea
						*Tomm22*	Chr15 : 79.670861	3.93	Cochlea
			rs46815278	0.36	Chr15 : 79.518120	*D730005E14Rik*	Chr15 : 79.889532	11.55	Cochlea
						*Cbx7*	Chr15 : 79.915807	2.89	Cochlea
								7.32	Cerebellum
						*Rpl3*	Chr15 : 80.077791	5.70	Cochlea
						*Dmc1*	Chr15 : 79.561497	2.88	Cochlea

Because Mendelian deafness genes frequently exhibit vestibular phenotypes [18–38], we performed a targeted evaluation of established deafness loci with documented vestibular involvement (e.g., *Myo7a*,* Slc26a4*,* Cdh23*) to assess whether they overlapped GWAS loci or demonstrated cis-eQTL support (Supplemental Table 5). None of these genes resided within the LD intervals of the lead GWAS SNPs, nor did they exhibit significant cis-eQTLs in the cochlear or cerebellar datasets analyzed. Consequently, these canonical Mendelian deafness genes were not prioritized under our regulatory-based candidate selection framework.

### cis-eQTL genes analysis in the cochlear tissue by utilizing human cochlea gene atlas and single-cell RNA sequencing data

**Fig. 5:**
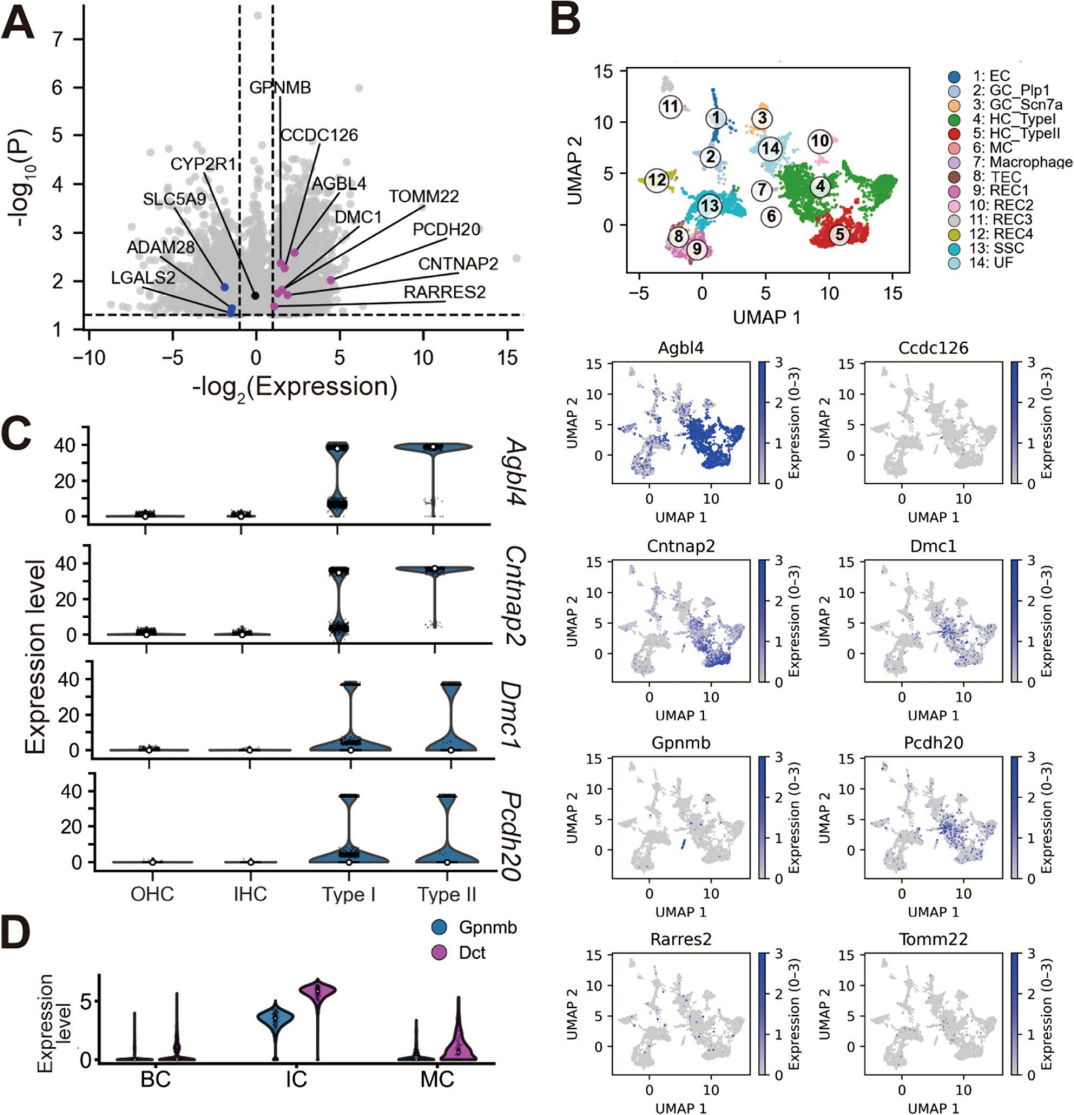
Expression profiles of candidate genes from vestibular GWAS loci. **A** Volcano plot showing overlap between mouse cochlear cis-eQTL genes and human cochlear-preferential genes. The x-axis indicates log₂ fold change (cochlea vs. 32 other human tissues) and the y-axis shows –log₁₀ P value. Magenta dots indicate cochlear-enriched genes (log₂FC > 1), black dots genes with moderate differences (–1 < log₂FC < 1), and blue dots vestibular-enriched genes (log₂FC < − 1). Dashed lines mark the thresholds for fold change (± 1) and significance. **A** Single-cell RNA-seq of the 3-month-old mouse utricle. The upper panel shows UMAP clusters, and the lower panels display feature plots of overlapping cis-eQTL genes. *Agbl4*, *Cntnap2*, *Dmc1*, and *Pcdh20* are enriched in hair-cell clusters, while *Gpnmb* is localized to the melanocyte cluster. Expression scale: 0–3. **B** Gene expression in hair-cell subtypes (OHC, IHC, type I, and type II) using 3-month-old mouse cochlea and utricle datasets. The y-axis shows log-transformed normalized counts per 10,000. All four genes are expressed in utricular hair cells, with *Agbl4* and *Cntnap2* showing higher expression in type II hair cells. **C** Expression of *Gpnmb* and *Dct* in the cochlear stria vascularis using 2-month-old mouse cochlea data. Gene expression is shown for basal cells (BC), intermediate cells (MC) and marginal cells (MC). *Gpnmb* is dominantly enriched in the intermediate cell

To further refine candidate selection, we compared the identified cis-eQTL genes with a publicly available atlas of human cochlear gene expression across 32 other tissues to identify cochlea-enriched genes [14]. Twelve of the 23 cis-eQTL genes showed preferential expression in the cochlea (Fig. 5A). We then examined these genes in publicly available single-nucleus RNA sequencing data from the mouse utricle [15] (Fig. 5B). Several, including *Agbl4*, *Cntnap2*, *Dmc1* and *Pcdh20* were expressed in HC clusters; *Agbl4* and *Cntnap2* were dominant in the type 2 HC (Fig. 5C). *Gpnmb* was specifically expressed in the melanocyte cluster. In the cochlea tissues, *Gpnmb* significantly expresses in the intermediate cells in the stria vascularis, supported by *Dct* of the well-known differentially expressed gene (DEG) of the intermediate cell [39, 40] (Fig. 5D).

## Discussion

We performed GWAS of VsEP and balance phenotypes across HMDP strains and identified several significant loci on chromosomes 4, 7, 14, and 15, as well as additional suggestive loci on chromosomes 6,7, and 14. To prioritize relevant candidate genes, we integrated cis-eQTLs data from GeneNetwork and identified 23 genes located within linkage disequilibrium intervals of the lead SNPs. Human cochlear expression profiles were then used to identify cochlea-preferentially expressed genes, followed by Single-cell RNA sequencing (scRNA-seq) analysis, which revealed *Agbl4*, *Cntnap2*, *Dmc1*, and *Pcdh20* are enriched in vestibular HC clusters, while *Gpnmb* is expressed in melanocytes. Importantly, the phenotypes analyzed represent continuous quantitative variation in vestibular physiology rather than binary loss-of-function states, supporting a polygenic model of vestibular regulation.

The HMDP continues to expand and is now widely applied in systems genetics studies across diverse phenotypes [41, 42]. In auditory research, recombinant inbred strains have enabled the identification of several loci and susceptible genes underlying hearing function and noise-induced hearing loss [43–46]. However, genetic studies on vestibular traits have been limited [11]. The only prior vestibular GWAS in mice identified *Dcc*, a gene required for peripheral vestibular innervation, using VsEP thresholds from 35 HMDP strains [47]. By increasing both the number of strains and phenotypic traits, our study successfully identified novel genes related to HCs and melanocytes function that contribute to vestibular and auditory physiology. Broad-sense heritability estimates ranging from 0.20 in young cohorts to 0.51 in aged males further support a measurable genetic contribution to vestibular function within this panel. Although approximately 500 individual animals were phenotyped, the effective unit of genetic mapping in the HMDP is the strain mean across 84 genetically distinct inbred lines. Replication within-strain reduces variance and increases the precision of strain means, thereby enhancing power to detect common variants of modest effect [48–50]. While allelic diversity in the HMDP is more limited than in highly outbred populations, this structured design enables high-resolution mapping of naturally segregating alleles under controlled environmental conditions. No significant loci were detected in the aged cohort, likely due to the challenges of maintaining and phenotyping frail aged mice [51, 52]. Nevertheless, the HMDP remains one of the most powerful and reproducible mouse resources for aging research because of its controlled environment, high resolution mapping, accessibility of relevant tissues, and integration with multi-omics data [53].

Because many Mendelian deafness genes exhibit vestibular phenotypes, we evaluated whether established deafness loci with documented vestibular involvement overlapped our GWAS intervals or demonstrated cis-eQTL support. None of these canonical genes resided within the LD blocks of the lead SNPs or showed significant cis-eQTL signals in the cochlear or cerebellar datasets analyzed. This distinction likely reflects fundamental differences between rare, high-penetrance coding mutations underlying Mendelian syndromes and the common regulatory variants segregating within the HMDP that contribute modest quantitative effects. Thus, the absence of classical deafness genes among our prioritized candidates is consistent with the polygenic, regulatory architecture interrogated in this study.

VsEP amplitude strongly reflects hair cell function [54], whereas the VsEP threshold is a multifactorial measure influenced by both sensory and neural components [55, 56]. *Agbl4* (ATP/GTP binding protein-like 4) encodes a deglutamylase enzyme involved in post-translational protein modification, though its cochlear role has not yet been defined. Notably, *Agbl4* is among the top 20 differentially expressed genes (DEGs) in mature utricular HCs from young to old [15], suggesting a potential role in vestibular hair cell maintenance. *Cntnap2*, identified from the young VsEP threshold GWAS, encodes a neuraxin-family adhesion molecule essential for neuronal signaling. *Cntnap2*-deficient rats show reduced auditory brainstem response amplitudes and delayed response latencies [57], and human GWAS have linked *CNTNAP2* to tinnitus and age-related hearing impairment [58], further supporting its role in inner ear function. *Pcdh20*, a member of the protocadherin family, is expressed in utricle hair cells [17], in organoid-derived hair cell from *Lgr5* + supporting cells [59], and in type 1 A spiral ganglion neurons [60]. *Dmc1* encodes a RecA-like recombinase essential for homologous chromosome synapsis and DNA repair during meiosis in germ cells. Disruption of *Dmc1* in mice leads to meiotic arrest and infertility [61], but there is no evidence of *Dmc1* expression or function in the cochlea or vestibular system. These findings underscore the critical importance of hair cells in vestibular performance and validate our GWAS and integrative bioinformatic approach for identifying vestibular-related genes. However, the precise function of these genes in the auditory and vestibular systems remain to be elucidated, and further experimental validation is essential.

Melanocytes, also termed perivascular-resident macrophage-like melanocytes (PVM/Ms), are distributed throughout the inner ear, including the stria vascularis, vestibular organs and endolymphatic sac, where they contribute to endolymph homeostasis [62–67]. In both humans and animal models, sensorineural hearing loss and vestibular disorders are associated with pigmentary abnormalities, either as syndromic manifestations or isolated symptoms [68–70]. PVM/Ms include the intermediate cells of the cochlear stria vascularis [71], and *Gpnmb* expression in this region is regulated by *Mitf*, a key melanocyte transcription factor [72]. Endolymphatic hydrops can alter vestibular transduction and VsEP response through mechanical or morphological changes in the labyrinth [73]. Thus, *Gpnmb* may influence vestibular function through both cochlear and vestibular melanocytes. Identification of *Gpnmb* from VsEP GWAS supports the importance of melanocyte function in vestibular physiology.

Several limitations should be noted. First, the modest sample size of 84 strains limits statistical power, particularly in the aged cohort, where smaller numbers of strain and mice were available. Second, strain overlap between young and aged cohorts was incomplete, and between-sex differences were not fully controlled, potentially contributing to phenotypic variability. Several traits showed elevated genomic inflation factors, suggesting that residual population structure or strain-specific effects may influence the test statics. In addition, characteristics of the phenotypes themselves may contribute to inflation ––for example, small numbers of mice per strain, variability in measurement reliability, non-normal distributions of vestibular traits, and the inherent difficulty of phenotyping aged or frail mice. These factors can increase phenotype-associated noise and elevate λGC values independent of genetic structure. Nonetheless, the biological relevance of the identified candidate genes is supported by independent single-cell transcriptomics evidence: these genes localize to specific cochlear and vestibular cell clusters reinforcing their functional plausibility despite statistical inflation. Furthermore, while pleiotropic effects cannot be fully excluded in complex-trait GWAS, our integrative prioritization using cis-eQTL data and cell-type-specific expression provides additional biological context that reduces the likelihood of purely spurious associations. Although cochlear and vestibular organs share common hair-cell machinery, prior analyses of inbred mouse panels have demonstrated that auditory brainstem response (ABR) phenotypes and vestibular measures exhibit distinct strain-dependent distributions [11, 43, 46], suggesting non-identical genetic architectures. Finaly, our analysis focused solely on SNPs, excluding indels, tandem repeats, and structural variants that might contribute to phenotypic variation.

Although direct gene-level replication with previously reported human vestibular GWAS loci was limited, several prioritized genes have independent experimental or genetic support in inner ear biology, strengthening their biological plausibility [7–10]. We further compared our loci with genes implicated in recent whole-exome and genome sequencing studies of Ménière’s disease and other vestibular disorders [74, 75]. No direct overlap was observed between genes highlighted in those sequencing analyses and loci identified in the present study. This lack of replication likely reflects differences in study design and genetic architecture: huma GWAS and sequencing studies interrogate heterogeneous clinical phenotypes and often emphasize rare coding variants or symptom-based traits, whereas our approach focuses on physiological measurements and common regulatory variation, providing a controlled experimental population. Future studies in independent cohorts and experimental systems will be necessary to replicate and functionally validate the loci identified here.

Together, our findings enhance understanding of the genetic underpinnings of vestibular function and cochlear physiology. The identification of genes in vestibular hair cell and melanocytes underscores the involvement of both sensory and non-sensory cell types in maintaining vestibular function. Although our results derive from murine data and require experimental validation, the integration of GWAS, eQTL, and single-cell transcriptomics provides a powerful framework for uncovering the molecular basis of balance.

## Conclusion

This study advances understanding of the genetic architecture underlying vestibular function and its decline with aging. Using VsEP and balance performance traits across HMDP strains, we identified several loci and candidate genes related to hair cell and melanocyte pathways essential for inner ear homeostasis. The discovery of *Agbl4*, *Cntnap2*, *Pcdh20*, *Dmc1*, and *Gpnmb* offers new insights into molecular mechanisms linking cellular degeneration in the otolithic organs to balance impairment in the elderly. While further validation is needed, these genes represent promising targets for mechanistic and translational studies aimed at preventing or mitigating age-related disequilibrium and fall risk.

## Material and method

### Animals

Recombinant inbred (RI) strains (BXD RI sets, derived from strains C57BL/6J and DBA/2J) and common inbred strains were obtained from the Jackson Laboratory (Bar Harbor, ME). For each HMDP strain, an average of six mice were used for VsEP and balance beam tests (Supplemental Tables 1 and 2). Because sex differences in vestibular function across ages have not been reported [14], only female mice were included in the young cohort. Mice arrived at 4 weeks of age and were acclimated to the animal facility for 2 weeks before testing at 6 weeks of age (total *n* = 504 for VsEP; *n* = 413 for the beam test). For the aged cohort, BXD mice were transferred from The University of Tennessee Health Science Center (UTHSC) and acclimated upon arrival. Enhanced monitoring, frailty assessment, husbandry and veterinary interventions were performed after 15 months of age [51]. The aged cohort included both sexes (males: 44 strains, *n* = 67; female: 63 strains, *n* = 145), and VsEP testing was performed at 22 ± 2.5 months of age (Supplemental Table 3). Mice were excluded if they exhibited health issues—such as tumors, dermatologic or ocular abnormalities, neurological signs, or other conditions for which the attending veterinarian recommended euthanasia based on frailty. For genetic analysis, male and female aged cohorts were analyzed separately in the GWAS to enable the detection of potential sex-specific genetic effects on vestibular function.

All animal protocols were approved (s17178) by the Institutional Care and Use Committee (IACUC) at University of California San Diego (UC San Diego).

### VsEP

VsEP Equipment and Acquisition VsEP recordings were based on methods detailed by Jones et al. [76]. Mice from each strain were weighed and anesthetized with an intraperitoneal injection of ketamine (100 mg/kg bodyweight) and xylazine (10 mg/kg bodyweight). Recording electrodes were placed subcutaneously at the nuchal crest (noninverting electrode), behind the right pinna (inverting electrode), and at the base of the tail (ground electrode). A noninvasive spring clip was placed on the head and secured to a voltage-controlled mechanical shaker. Linear acceleration pulses lasting 2 ms each were applied to the cranium in the naso-occipital axis at a rate of 17 pulses per second. Pulses were presented using two polarities: normal (+ Gx axis) and inverted (− Gx axis). Stimulus amplitudes ranged from + 6 to − 18 dB re: 1 g/ms (1.0 g = 9.8 m/s2) and were presented in steps of 3 dB. VsEPs were recorded using traditional signal averaging. Electrophysiological activity was amplified (200,000×), filtered (300 to 3000 Hz), and digitized (5,000 Hz) beginning at stimulus onset. Responses (*n* = 256) were averaged to produce one response trace and replicated to provide at least two sets of waveform averages at each stimulus intensity. Waveforms were collected with and without the presence of broadband forward masker (50–50,000 Hz, 90 dB SPL). VsEP intensity series was collected beginning at the lowest stimulus intensity (− 18 dB re: 1.0 g/ms) with and without acoustic masking, then in ascending 3 dB steps to + 6 dB re: 1.0 g/ms. The first two positive and negative response peaks were analyzed. Peak-to-peak amplitudes were measured in microvolts from each positive response peak (P1 or P2) to the respective negative response peak (N1 or N2). Threshold (measured in dB re: 1.0 g/ms) was defined as the stimulus level midway between the jerk amplitude producing a discernible response and the stimulus level which did not. ABR Peak Analysis Software Version 0.9.0.2 ©Copyright 2007 Speech and Hearing Bioscience and Technology was used to analyze VsEP waveforms.

### Balance beam test

Vestibulo-motor function was assessed using an elevated using elevated balance beam assay. Mice traversed a round plastic bar (15 mm in diameter, 60 cm in length, ) inclined at 5.3°. The traversal time was recorded with a 30 s cutoff. To facilitate task acquisition, enclosed safe boxes were placed at the beam end. Prior to testing, mice received brief training trials until they could reliably complete the task. Mice were considered reliable when they could traverse the beam without pausing or turning back within 30 s. Each mouse subsequently performed three test trials separated by 5-min intervals. All crossings were video recorded for quantitative analysis.

### GWAS analysis

Association Analysis GWAS analyses for vestibular phenotypes in the HMDP strains were performed using genotypes of 459,911 SNPs obtained from the Mouse Diversity Array [77]. SNPs were required to have minor allele frequencies ≥ 5% and missing genotype frequencies ≤ 10% within the phenotyped strains for each trait analyzed. After applying these filtering criteria, the final set of SNPs used for analysis varied from ~ 104,000-166,000 depending on the exact strain composition of the phenotyped mice (Supplemental Table 4). All phenot ypic measures were log10-transformed prior to association testing to improve normality and stabilize variance across strains. Association testing was performed using FaST-LMM [78], a linear mixed model method that is fast and accounts for potential cofounding variables like population structure. To improve power, when testing all SNPs on a specific chromosome, the kinship matrix was constructed using the SNPs from all other chromosomes [79, 80]. This procedure includes the SNP being tested for association in the regression equation only once. Genome-wide significance threshold in the HMDP was determined by the family-wise error rate (FWER) as the probability of observing one or more false positives across all SNPs per phenotype. We ran 100 different sets of permutation tests and parametric bootstrapping of size 1000 and observed that the genome-wide significance threshold at a FWER of 0.05 corresponded to -log(p) = 4.1 × 10^6^, similar to what has been used in previous studies with the HMDP [13]. This is approximately an order of magnitude larger than the threshold obtained by Bonferroni correction (-log(p) = 4.6 × 10^7^), which would be an overly conservative estimate of significance because nearby SNPs among inbred mouse strains are not independent, instead, they are highly correlated with each other. Nonsynonymous SNPs within each coding region were downloaded from the Mouse Phenome Database (http://phenome.jax.org/). Regional plots were generated with a standalone version of LocusZoom 1.4 [81].

### Heritability estimation

Broad-sense heritability (H²) was estimated using linear mixed-effects model implemented in Python 3.10 using the statsmodels package. To partition variance, strain was included as a random effect, and the model was fitted using restricted maximum likelihood (REML). Variance components were extracted to calculate heritability using the following formula:

$$\:{H}^{2}=\frac{{\sigma\:}_{G}^{2}}{{\sigma\:}_{G}^{2}+{\sigma\:}_{E}^{2}}$$

Because HMDP strains are fully inbred, the between-strain variance ($\:\:{\sigma\:}_{G}^{2}\:$) represents total genetic valiation, whereas the within-strain variance ($\:{\sigma\:}_{E}^{2}$) primarily reflects environmental and measurement variation. *H*^*2*^ was calculated separately for each phenotype and age group.

### cis-eQTL analysis

cis-eQTL mapping was performed using GeneNetwork (genenetwork.org) within the BXD recombinant inbred mouse panel; BXD strains make up a portion of the HMDP. Two datasets were analyzed: (i) UTHSC BXD Cochlea 2–8 wks RNA-Seq (Jun25) TPM Log2 and (ii) SJUT Cerebellum mRNA M430 (Oct04) PDNN. For each GWAS lead SNP, we defined a cis window of ± 2 Mb around the GWAS peak position and examined all genes within the LD region (r^2^ > 0.8). Mapping was conducted using GEMMA, applying a linear mixed model with kinship correction to account for genetic relatedness among strains. Genes with a cis-eQTL q-value < 0.05 were considered significant and prioritized as candidate regulatory genes at the corresponding GWAS loci.

### Single-cell RNA sequencing (scRNA-seq) analysis

Single-cell and single-nucleus RNA sequencing datasets from mouse utricle and cochlea were obtained from the Gene Expression Omnibus (https://www.ncbi.nlm.nih.gov/geo/) (GSE274279) [15] and the gEAR portal (https://umgear.org/index.html) [82–84]. Data processing was performed using Scanpy in Python [85]. Low-quality cells (< 200 genes or > 5% mitochondrial unique molecular identifier (UMIs)) were excluded, and counts were normalized to 10,000 per cell and log-transformed. Dimensionality reduction was conducted using principal component analysis (PCA), followed by Leiden clustering (resolution = 0.5) and UMAP visualization. Cell types were annotated based on established marker genes reported in the original studies [16–18, 21].

## Supplementary Information

Supplementary Material 1.

Supplementary Material 2.

Supplementary Material 3.

Supplementary Material 4.

Supplementary Material 5.

Supplementary Material 6.

## Data Availability

The human cochlear transcriptomic dataset used in this study was originally reported by Schrauwen et al. [14]. As the original download link provided in that publication is no longer active, the dataset has been redeposited in Figshare with permission from the corresponding author and is available at 10.6084/m9.figshare.31668889. The GWAS summary statics generated during the current study are available in Figshare at: 10.6084/m9.figshare.31840561. The single-cell and single-nucleus RNA sequencing datasets analyzed in this study are publicly available: • Mouse utricle snRNA-seq data (GEO: GSE274279) at https://www.ncbi.nlm.nih.gov/geo/query/acc.cgi?acc=GSE274279 Mouse cochlear stria dataset hosted on the gEAR portal at https://umgear.org/p?s=96e1b4f9 Cis-eQTL datasets from GeneNetwork used to evaluate gene regulation at GWAS-associated loci are publicly accessible: • UTHSC BXD Cochlea 2–8 wks RNA-Seq (Jun25) TPM Log2 dataset: https://info.genenetwork.org/infofile/source.php?GN_AccesionId=1074 • SJUT Cerebellum mRNA M430 (Oct04) PDNN dataset: https://info.genenetwork.org/infofile/source.php?GN_AccesionId=44 Phenotypic data for all VsEP and raised-beam measurements are provided in the Supplementary Tables of this article.

## References

[1] Barin K, Dodson EE. Dizziness in the elderly. Otolaryngol Clin North Am. 2011;44:437–54. 10.1016/j.otc.2011.01.013. x. 21474016 10.1016/j.otc.2011.01.013

[2] Jönsson R, Sixt E, Landahl S, Rosenhall U. Prevalence of dizziness and vertigo in an urban elderly population. J Vestib Res. 2004;14:47–52. 15156096

[3] Peterson AB, Kegler SR. Deaths from Fall-Related Traumatic Brain Injury - United States, 2008–2017. MMWR Morb Mortal Wkly Rep. 2020;69:225–30. 10.15585/mmwr.mm6909a2. 32134910 10.15585/mmwr.mm6909a2 PMC7367089

[4] Trajanoska K, Seppala LJ, Medina-Gomez C, Hsu Y-H, Zhou S, van Schoor NM, et al. Genetic basis of falling risk susceptibility in the UK Biobank Study. Commun Biol. 2020;3:543. 10.1038/s42003-020-01256-x. 32999390 10.1038/s42003-020-01256-x PMC7527955

[5] Gabriel GA, Harris LR, Gnanasegaram JJ, Cushing SL, Gordon KA, Haycock BC, et al. Age-related changes to vestibular heave and pitch perception and associations with postural control. Sci Rep Nat Publishing Group. 2022;12:6426. 10.1038/s41598-022-09807-4. 10.1038/s41598-022-09807-4 PMC9018785 35440744

[6] Ueda K, Imai T, Ito T, Okayasu T, Harada S, Kamakura T, et al. Effects of aging on otolith morphology and functions in mice. Front Neurosci. 2024;18:1466514. 10.3389/fnins.2024.1466514. 39479359 10.3389/fnins.2024.1466514 PMC11521974

[7] Skuladottir AT, Bjornsdottir G, Nawaz MS, Petersen H, Rognvaldsson S, Moore KHS, et al. A genome-wide meta-analysis uncovers six sequence variants conferring risk of vertigo. Commun Biol Nat Publishing Group. 2021;4:1148. 10.1038/s42003-021-02673-2. 10.1038/s42003-021-02673-2 PMC8497462 34620984

[8] Rujescu D, Hartmann AM, Giegling I, Konte B, Herrling M, Himmelein S, et al. Genome-Wide Association Study in Vestibular Neuritis: Involvement of the Host Factor for HSV-1 Replication. Front Neurol. 2018;9:591. 10.3389/fneur.2018.00591. 30079052 10.3389/fneur.2018.00591 PMC6062961

[9] Hromatka BS, Tung JY, Kiefer AK, Do CB, Hinds DA, Eriksson N. Genetic variants associated with motion sickness point to roles for inner ear development, neurological processes and glucose homeostasis. Hum Mol Genet. 2015;24:2700–8. 10.1093/hmg/ddv028. 25628336 10.1093/hmg/ddv028 PMC4383869

[10] Clifford R, Munro D, Dochtermann D, Devineni P, Pyarajan S, Telese F, et al. Genome-Wide Association Study of Chronic Dizziness in the Elderly Identifies Loci Implicating MLLT10, BPTF, LINC01224, and ROS1. J Assoc Res Otolaryngol. 2023;24:575–91. 10.1007/s10162-023-00917-y. 38036714 10.1007/s10162-023-00917-y PMC10752854

[11] Salehi P, Myint A, Kim YJ, Ge MX, Lavinsky J, Ho MK, et al. Genome-Wide Association Analysis Identifies Dcc as an Essential Factor in the Innervation of the Peripheral Vestibular System in Inbred Mice. J Assoc Res Otolaryngol. 2016;17:417–31. 10.1007/s10162-016-0578-4. 27539716 10.1007/s10162-016-0578-4 PMC5023540

[12] Honaker JA, Lee C, Criter RE, Jones TA. Test-retest reliability of the vestibular sensory-evoked potential (VsEP) in C57BL/6J mice. J Am Acad Audiol. 2015;26:59–67. 10.3766/jaaa.26.1.7. 25597461 10.3766/jaaa.26.1.7

[13] Ghazalpour A, Rau CD, Farber CR, Bennett BJ, Orozco LD, van Nas A, et al. Hybrid mouse diversity panel: a panel of inbred mouse strains suitable for analysis of complex genetic traits. Mamm Genome. 2012;23:680–92. 10.1007/s00335-012-9411-5. 22892838 10.1007/s00335-012-9411-5 PMC3586763

[14] Schrauwen I, Hasin-Brumshtein Y, Corneveaux JJ, Ohmen J, White C, Allen AN, et al. A comprehensive catalogue of the coding and non-coding transcripts of the human inner ear. Hear Res. 2016;333:266–74. 10.1016/j.heares.2015.08.013. 26341477 10.1016/j.heares.2015.08.013 PMC4775449

[15] Xia M, Zhang F, Ma J, Li Y, Jia G, Wu M, et al. Single-nucleus profiling of mouse inner ear aging uncovers cell type heterogeneity and hair cell subtype-specific age-related signatures. Cell Rep. 2025;44:115781. 10.1016/j.celrep.2025.115781. 40440168 10.1016/j.celrep.2025.115781

[16] Schoen CJ, Burmeister M, Lesperance MM. Diaphanous homolog 3 (Diap3) overexpression causes progressive hearing loss and inner hair cell defects in a transgenic mouse model of human deafness. PLoS ONE. 2013;8:e56520. 10.1371/journal.pone.0056520. 23441200 10.1371/journal.pone.0056520 PMC3575478

[17] Vuckovic D, Dawson S, Scheffer DI, Rantanen T, Morgan A, Di Stazio M, et al. Genome-wide association analysis on normal hearing function identifies PCDH20 and SLC28A3 as candidates for hearing function and loss. Hum Mol Genet. 2015;24:5655–64. 10.1093/hmg/ddv279. 26188009 10.1093/hmg/ddv279 PMC4572074

[18] Kharkovets T, Hardelin JP, Safieddine S, Schweizer M, El-Amraoui A, Petit C, et al. KCNQ4, a K+ channel mutated in a form of dominant deafness, is expressed in the inner ear and the central auditory pathway. Proc Natl Acad Sci U S A. 2000;97:4333–8. 10.1073/pnas.97.8.4333. 10760300 10.1073/pnas.97.8.4333 PMC18242

[19] Wesdorp M, de Koning Gans PAM, Schraders M, Oostrik J, Huynen MA, Venselaar H, et al. Heterozygous missense variants of LMX1A lead to nonsyndromic hearing impairment and vestibular dysfunction. Hum Genet. 2018;137:389–400. 10.1007/s00439-018-1880-5. 29754270 10.1007/s00439-018-1880-5 PMC5973959

[20] Robertson NG, Lu L, Heller S, Merchant SN, Eavey RD, McKenna M, et al. Mutations in a novel cochlear gene cause DFNA9, a human nonsyndromic deafness with vestibular dysfunction. Nat Genet. 1998;20:299–303. 10.1038/3118. 9806553 10.1038/3118

[21] Weil D, Küssel P, Blanchard S, Lévy G, Levi-Acobas F, Drira M, et al. The autosomal recessive isolated deafness, DFNB2, and the Usher 1B syndrome are allelic defects of the myosin-VIIA gene. Nat Genet. 1997;16:191–3. 10.1038/ng0697-191. 9171833 10.1038/ng0697-191

[22] Bahloul A, Michel V, Hardelin J-P, Nouaille S, Hoos S, Houdusse A, et al. Cadherin-23, myosin VIIa and harmonin, encoded by Usher syndrome type I genes, form a ternary complex and interact with membrane phospholipids. Hum Mol Genet. 2010;19:3557–65. 10.1093/hmg/ddq271. 20639393 10.1093/hmg/ddq271 PMC2928128

[23] McGuirt WT, Prasad SD, Griffith AJ, Kunst HP, Green GE, Shpargel KB, et al. Mutations in COL11A2 cause non-syndromic hearing loss (DFNA13). Nat Genet. 1999;23:413–9. 10.1038/70516. 10581026 10.1038/70516

[24] Lalwani AK, Goldstein JA, Kelley MJ, Luxford W, Castelein CM, Mhatre AN. Human nonsyndromic hereditary deafness DFNA17 is due to a mutation in nonmuscle myosin MYH9. Am J Hum Genet. 2000;67:1121–8. 10.1016/S0002-9297(07)62942-5. 11023810 10.1016/s0002-9297(07)62942-5 PMC1288554

[25] Seco CZ, Oonk AMM, Domínguez-Ruiz M, Draaisma JMT, Gandía M, Oostrik J, et al. Progressive hearing loss and vestibular dysfunction caused by a homozygous nonsense mutation in CLIC5. Eur J Hum Genet. 2015;23:189–94. 10.1038/ejhg.2014.83. 24781754 10.1038/ejhg.2014.83 PMC4297911

[26] Schraders M, Oostrik J, Huygen PLM, Strom TM, van Wijk E, Kunst HPM, et al. Mutations in PTPRQ Are a Cause of Autosomal-Recessive Nonsyndromic Hearing Impairment DFNB84 and Associated with Vestibular Dysfunction. Am J Hum Genet Elsevier. 2010;86:604–10. 10.1016/j.ajhg.2010.02.015. 10.1016/j.ajhg.2010.02.015 PMC2850434 20346435

[27] de Kok YJ, van der Maarel SM, Bitner-Glindzicz M, Huber I, Monaco AP, Malcolm S, et al. Association between X-linked mixed deafness and mutations in the POU domain gene POU3F4. Volume 267. New York, N.Y.: Science; 1995. pp. 685–8. 10.1126/science.7839145. 10.1126/science.7839145 7839145

[28] Ahmed ZM, Smith TN, Riazuddin S, Makishima T, Ghosh M, Bokhari S, et al. Nonsyndromic recessive deafness DFNB18 and Usher syndrome type IC are allelic mutations of USHIC. Hum Genet. 2002;110:527–31. 10.1007/s00439-002-0732-4. 12107438 10.1007/s00439-002-0732-4

[29] Alagramam KN, Yuan H, Kuehn MH, Murcia CL, Wayne S, Srisailpathy CR, et al. Mutations in the novel protocadherin PCDH15 cause Usher syndrome type 1F. Hum Mol Genet. 2001;10:1709–18. 10.1093/hmg/10.16.1709. 11487575 10.1093/hmg/10.16.1709

[30] Ebermann I, Walger M, Scholl HPN, Charbel Issa P, Lüke C, Nürnberg G, et al. Truncating mutation of the DFNB59 gene causes cochlear hearing impairment and central vestibular dysfunction. Hum Mutat. 2007;28:571–7. 10.1002/humu.20478. 17301963 10.1002/humu.20478

[31] Rohacek AM, Bebee TW, Tilton RK, Radens CM, McDermott-Roe C, Peart N, et al. ESRP1 Mutations Cause Hearing Loss due to Defects in Alternative Splicing that Disrupt Cochlear Development. Dev Cell. 2017;43:318–e3315. 10.1016/j.devcel.2017.09.026. 29107558 10.1016/j.devcel.2017.09.026 PMC5687886

[32] Schraders M, Ruiz-Palmero L, Kalay E, Oostrik J, del Castillo FJ, Sezgin O, et al. Mutations of the gene encoding otogelin are a cause of autosomal-recessive nonsyndromic moderate hearing impairment. Am J Hum Genet. 2012;91:883–9. 10.1016/j.ajhg.2012.09.012. 23122587 10.1016/j.ajhg.2012.09.012 PMC3487128

[33] Tsukada K, Nishio S, Takumi Y, Usami S. Comparison of vestibular function in hereditary hearing loss patients with GJB2, CDH23, and SLC26A4 variants. Sci Rep Nat Publishing Group. 2024;14:10596. 10.1038/s41598-024-61442-3. 10.1038/s41598-024-61442-3 PMC11078969 38720048

[34] Verpy E, Masmoudi S, Zwaenepoel I, Leibovici M, Hutchin TP, Del Castillo I, et al. Mutations in a new gene encoding a protein of the hair bundle cause non-syndromic deafness at the DFNB16 locus. Nat Genet. 2001;29:345–9. 10.1038/ng726. 11687802 10.1038/ng726

[35] Verpy E, Weil D, Leibovici M, Goodyear RJ, Hamard G, Houdon C, et al. Stereocilin-deficient mice reveal the origin of cochlear waveform distortions. Nature. 2008;456:255–8. 10.1038/nature07380. 18849963 10.1038/nature07380 PMC3338146

[36] Odeh H, Hunker KL, Belyantseva IA, Azaiez H, Avenarius MR, Zheng L, et al. Mutations in Grxcr1 Are The Basis for Inner Ear Dysfunction in the Pirouette Mouse. Am J Hum Genet. 2010;86:148–60. 10.1016/j.ajhg.2010.01.016. 20137774 10.1016/j.ajhg.2010.01.016 PMC2820167

[37] Diaz-Horta O, Abad C, Sennaroglu L, Foster J, DeSmidt A, Bademci G, et al. ROR1 is essential for proper innervation of auditory hair cells and hearing in humans and mice. Proc Natl Acad Sci U S A. 2016;113:5993–8. 10.1073/pnas.1522512113. 27162350 10.1073/pnas.1522512113 PMC4889368

[38] Donaudy F, Zheng L, Ficarella R, Ballana E, Carella M, Melchionda S, et al. Espin gene (ESPN) mutations associated with autosomal dominant hearing loss cause defects in microvillar elongation or organisation. J Med Genet. 2006;43:157–61. 10.1136/jmg.2005.032086. 15930085 10.1136/jmg.2005.032086 PMC2564636

[39] Jean P, Wong Jun Tai F, Singh-Estivalet A, Lelli A, Scandola C, Megharba S, et al. Single-cell transcriptomic profiling of the mouse cochlea: An atlas for targeted therapies. Proc Natl Acad Sci U S A. 2023;120:e2221744120. 10.1073/pnas.2221744120. 37339214 10.1073/pnas.2221744120 PMC10293812

[40] Sun G, Zheng Y, Fu X, Zhang W, Ren J, Ma S, et al. Single-cell transcriptomic atlas of mouse cochlear aging. Protein Cell. 2022;14:180–201. 10.1093/procel/pwac058. 10.1093/procel/pwac058 PMC10098046 36933008

[41] Fang Z, Peltz G. Twenty-first century mouse genetics is again at an inflection point. Lab Anim Nat Publishing Group. 2025;54:9–15. 10.1038/s41684-024-01491-3. 10.1038/s41684-024-01491-3 PMC11695262 39592878

[42] Jurrjens AW, Seldin MM, Giles C, Meikle PJ, Drew BG, Calkin AC. The potential of integrating human and mouse discovery platforms to advance our understanding of cardiometabolic diseases. James DE, editor. eLife. eLife Sciences Publications, Ltd; 2023;12:e86139. 10.7554/eLife.86139. 10.7554/eLife.86139 PMC10065800 37000167

[43] Crow AL, Ohmen J, Wang J, Lavinsky J, Hartiala J, Li Q et al. The Genetic Architecture of Hearing Impairment in Mice: Evidence for Frequency-Specific Genetic Determinants. G3 Genes|Genomes|Genetics. 2015;5:2329–39. 10.1534/g3.115.021592. 10.1534/g3.115.021592 PMC4632053 26342000

[44] Boussaty EC, Gillard D, Lavinsky J, Salehi P, Wang J, Mendonça A, et al. The Genetics of Variation of the Wave 1 Amplitude of the Mouse Auditory Brainstem Response. JARO. 2020;21:323–36. 10.1007/s10162-020-00762-3. 32757112 10.1007/s10162-020-00762-3 PMC7445221

[45] Ohmen J, Kang EY, Li X, Joo JW, Hormozdiari F, Zheng QY, et al. Genome-Wide Association Study for Age-Related Hearing Loss (AHL) in the Mouse: A Meta-Analysis. J Assoc Res Otolaryngol. 2014;15:335–52. 10.1007/s10162-014-0443-2. 24570207 10.1007/s10162-014-0443-2 PMC4010595

[46] Lavinsky J, Crow AL, Pan C, Wang J, Aaron KA, Ho MK, et al. Genome-Wide Association Study Identifies Nox3 as a Critical Gene for Susceptibility to Noise-Induced Hearing Loss. PLOS Genet Public Libr Sci. 2015;11:e1005094. 10.1371/journal.pgen.1005094. 10.1371/journal.pgen.1005094 PMC4399881 25880434

[47] Salehi P, Myint A, Kim YJ, Ge MX, Lavinsky J, Ho MK, et al. Genome-Wide Association Analysis Identifies Dcc as an Essential Factor in the Innervation of the Peripheral Vestibular System in Inbred Mice. JARO. 2016;17:417–31. 10.1007/s10162-016-0578-4. 27539716 10.1007/s10162-016-0578-4 PMC5023540

[48] Belknap JK. Effect of within-strain sample size on QTL detection and mapping using recombinant inbred mouse strains. Behav Genet. 1998;28:29–38. 10.1023/a:1021404714631. 9573644 10.1023/a:1021404714631

[49] Valdar W, Flint J, Mott R. Simulating the Collaborative Cross: Power of Quantitative Trait Loci Detection and Mapping Resolution in Large Sets of Recombinant Inbred Strains of Mice. Genetics. 2006;172:1783–97. 10.1534/genetics.104.039313. 16361245 10.1534/genetics.104.039313 PMC1456308

[50] Keele GR, Crouse WL, Kelada SNP, Valdar W. Determinants of QTL Mapping Power in the Realized Collaborative Cross. G3 Genes Genomes Genetics. 2019;9:1707–27. 10.1534/g3.119.400194. 10.1534/g3.119.400194 PMC6505132 30914424

[51] Wilkinson MJ, Selman C, McLaughlin L, Horan L, Hamilton L, Gilbert C, et al. Progressing the care, husbandry and management of ageing mice used in scientific studies. Lab Anim. 2020;54:225–38. 10.1177/0023677219865291. 31403890 10.1177/0023677219865291 PMC7301645

[52] Malavolta M. Anti-aging interventions in geriatric mice: insights into the timing of treatment, benefits, and limitations. Geroscience. 2025;47:109–19. 10.1007/s11357-024-01309-7. 39112719 10.1007/s11357-024-01309-7 PMC11872812

[53] Li H, Auwerx J. Mouse Systems Genetics as a Prelude to Precision Medicine. Trends Genet. 2020;36:259–72. 10.1016/j.tig.2020.01.004. 32037011 10.1016/j.tig.2020.01.004 PMC7106150

[54] Ratzan EM, Lee J, Madison MA, Zhu H, Zhou W, Géléoc GSG, et al. TMC function, dysfunction, and restoration in mouse vestibular organs. Front Neurol. 2024;15:1356614. 10.3389/fneur.2024.1356614. 38638308 10.3389/fneur.2024.1356614 PMC11024474

[55] Jones SM, Johnson KR, Yu H, Erway LC, Alagramam KN, Pollak N, et al. A Quantitative Survey of Gravity Receptor Function in Mutant Mouse Strains. JARO. 2005;6:297–310. 10.1007/s10162-005-0009-4. 16235133 10.1007/s10162-005-0009-4 PMC2504620

[56] Wan G, Ji L, Schrepfer T, Gong S, Wang G-P, Corfas G. Synaptopathy as a Mechanism for Age-Related Vestibular Dysfunction in Mice. Front Aging Neurosci. 2019;11:156. 10.3389/fnagi.2019.00156. 31293415 10.3389/fnagi.2019.00156 PMC6606700

[57] Scott KE, Schormans AL, Pacoli KY, Oliveira CD, Allman BL, Schmid S. Altered Auditory Processing, Filtering, and Reactivity in the Cntnap2 Knock-Out Rat Model for Neurodevelopmental Disorders. J Neurosci Soc Neurosci. 2018;38:8588–604. 10.1523/JNEUROSCI.0759-18.2018. 10.1523/JNEUROSCI.0759-18.2018 PMC6596223 30126973

[58] Fransen E, Bonneux S, Corneveaux JJ, Schrauwen I, Di Berardino F, White CH, et al. Genome-wide association analysis demonstrates the highly polygenic character of age-related hearing impairment. Eur J Hum Genet. 2015;23:110–5. 10.1038/ejhg.2014.56. 24939585 10.1038/ejhg.2014.56 PMC4266741

[59] Kalra G, Lenz D, Abdul-Aziz D, Hanna C, Basu M, Herb BR, et al. Cochlear organoids reveal transcriptional programs of postnatal hair cell differentiation from supporting cells. Cell Rep. 2023;42:113421. 10.1016/j.celrep.2023.113421. 37952154 10.1016/j.celrep.2023.113421 PMC11007545

[60] Shrestha BR, Chia C, Wu L, Kujawa SG, Liberman MC, Goodrich LV. Sensory Neuron Diversity in the Inner Ear Is Shaped by Activity. Cell Elsevier. 2018;174:1229–e124617. 10.1016/j.cell.2018.07.007. 10.1016/j.cell.2018.07.007 PMC6150604 30078709

[61] Yoshida K, Kondoh G, Matsuda Y, Habu T, Nishimune Y, Morita T. The Mouse *RecA*-like Gene *Dmc1* Is Required for Homologous Chromosome Synapsis during Meiosis. Mol Cell. 1998;1:707–18. 10.1016/S1097-2765(00)80070-2. 9660954 10.1016/s1097-2765(00)80070-2

[62] Cable J, Steel KP. Identification of two types of melanocyte within the stria vascularis of the mouse inner ear. Pigment Cell Res. 1991;4:87–101. 10.1111/j.1600-0749.1991.tb00320.x. 1946214 10.1111/j.1600-0749.1991.tb00320.x

[63] Ciuman RR. Stria vascularis and vestibular dark cells: characterisation of main structures responsible for inner-ear homeostasis, and their pathophysiological relations. J Laryngol Otol. 2009;123:151–62. 10.1017/S0022215108002624. 18570690 10.1017/S0022215108002624

[64] Conlee JW, Parks TN, Schwartz IR, Creel DJ. Comparative anatomy of melanin pigment in the stria vascularis. Evidence for a distinction between melanocytes and intermediate cells in the cat. Acta Otolaryngol. 1989;107:48–58. 10.3109/00016488909127478. 2929316 10.3109/00016488909127478

[65] Escobar C, Zuasti A, Ferrer C, Garcia-Ortega F. Melanocytes in the stria vascularis and vestibular labyrinth of the Mongolian gerbil (Meriones unguiculatus). Pigment Cell Res. 1995;8:271–8. 10.1111/j.1600-0749.1995.tb00674.x. 8789202 10.1111/j.1600-0749.1995.tb00674.x

[66] van Beelen ESA, van der Valk WH, de Groot JCMJ, Hensen EF, Locher H, van Benthem PPG. Migration and fate of vestibular melanocytes during the development of the human inner ear. Dev Neurobiol. 2020;80:411–32. 10.1002/dneu.22786. 33075185 10.1002/dneu.22786 PMC7894185

[67] Zhang W, Dai M, Fridberger A, Hassan A, DeGagne J, Neng L et al. Perivascular-resident macrophage-like melanocytes in the inner ear are essential for the integrity of the intrastrial fluid–blood barrier. Proceedings of the National Academy of Sciences. Proc Natl Acad Sci. 2012;109:10388–93. 10.1073/pnas.1205210109 22689949 10.1073/pnas.1205210109 PMC3387119

[68] Lavezzo MM, Sakata VM, Morita C, Rodriguez EEC, Abdallah SF, da Silva FTG, et al. Vogt-Koyanagi-Harada disease: review of a rare autoimmune disease targeting antigens of melanocytes. Orphanet J Rare Dis. 2016;11:29. 10.1186/s13023-016-0412-4. 27008848 10.1186/s13023-016-0412-4 PMC4806431

[69] Pingault V, Ente D, Dastot-Le Moal F, Goossens M, Marlin S, Bondurand N. Review and update of mutations causing Waardenburg syndrome. Hum Mutat. 2010;31:391–406. 10.1002/humu.21211. 20127975 10.1002/humu.21211

[70] Barozzi S, Ginocchio D, Socci M, Alpini D, Cesarani A. Audiovestibular disorders as autoimmune reaction in patients with melanoma. Med Hypotheses. 2015;85:336–8. 10.1016/j.mehy.2015.06.009. 26115944 10.1016/j.mehy.2015.06.009

[71] Gu X, Jiang K, Chen R, Chen Z, Wu X, Xiang H, et al. Identification of common stria vascularis cellular alteration in sensorineural hearing loss based on ScRNA-seq. BMC Genomics. 2024;25:213. 10.1186/s12864-024-10122-7. 38413848 10.1186/s12864-024-10122-7 PMC10897997

[72] Chen L, Wang L, Chen L, Wang F, Ji F, Sun W, et al. Transcript Profiles of Stria Vascularis in Models of Waardenburg Syndrome. Neural Plast. 2020;2020:2908182. 10.1155/2020/2908182. 32802035 10.1155/2020/2908182 PMC7416267

[73] Pastras CJ, Stefani SP, Curthoys IS, Camp AJ, Brown DJ. Utricular Sensitivity during Hydrodynamic Displacements of the Macula. J Assoc Res Otolaryngol. 2020;21:409–23. 10.1007/s10162-020-00769-w. 32783163 10.1007/s10162-020-00769-w PMC7567774

[74] Parra-Perez AM, Gallego-Martinez A, Escalera-Balsera A, Robles-Bolivar P, Perez-Carpena P, Lopez-Escamez JA. Different Contribution of Missense and Loss-of-Function Variants to the Genetic Structure of Familial and Sporadic Meniere Disease. MedComm. 2025;6:e70394. . 10.1002/mco2.70394 40989574 10.1002/mco2.70394 PMC12450834

[75] Fisch KM, Rosenthal SB, Mark A, Sasik R, Nasamran CA, Clifford R, et al. The genomic landscape of Ménière’s disease: a path to endolymphatic hydrops. BMC Genomics. 2024;25:646. 10.1186/s12864-024-10552-3. 38943082 10.1186/s12864-024-10552-3 PMC11212243

[76] Jones TA, Jones SM, Vijayakumar S, Brugeaud A, Bothwell M, Chabbert C. The adequate stimulus for mammalian linear vestibular evoked potentials (VsEPs). Hear Res. 2011;280. 10.1016/j.heares.2011.05.005. 10.1016/j.heares.2011.05.005 PMC3826178 21664446

[77] Yang H, Ding Y, Hutchins LN, Szatkiewicz J, Bell TA, Paigen B, et al. A customized and versatile high-density genotyping array for the mouse. Nat Methods. 2009;6:663–6. 10.1038/nmeth.1359. 19668205 10.1038/nmeth.1359 PMC2735580

[78] Lippert C, Listgarten J, Liu Y, Kadie CM, Davidson RI, Heckerman D. FaST linear mixed models for genome-wide association studies. Nat Methods. 2011;8:833–5. 10.1038/nmeth.1681. 21892150 10.1038/nmeth.1681

[79] Listgarten J, Lippert C, Kadie CM, Davidson RI, Eskin E, Heckerman D. Improved linear mixed models for genome-wide association studies. Nat Methods. 2012;9:525–6. 10.1038/nmeth.2037. 22669648 10.1038/nmeth.2037 PMC3597090

[80] Cheng R, Parker CC, Abney M, Palmer AA. Practical considerations regarding the use of genotype and pedigree data to model relatedness in the context of genome-wide association studies. G3 (Bethesda). 2013;3:1861–7. 10.1534/g3.113.007948. 23979941 10.1534/g3.113.007948 PMC3789811

[81] Pruim RJ, Welch RP, Sanna S, Teslovich TM, Chines PS, Gliedt TP, et al. LocusZoom: regional visualization of genome-wide association scan results. Bioinformatics. 2010;26:2336–7. 10.1093/bioinformatics/btq419. 20634204 10.1093/bioinformatics/btq419 PMC2935401

[82] Sun G, Zheng Y, Fu X, Zhang W, Ren J, Ma S, et al. Single-cell transcriptomic atlas of mouse cochlear aging. Protein Cell. 2023;14:180–201. 10.1093/procel/pwac058. 36933008 10.1093/procel/pwac058 PMC10098046

[83] Boussaty EC, Tedeschi N, Novotny M, Ninoyu Y, Du E, Draf C, et al. Cochlear transcriptome analysis of an outbred mouse population (CFW). Front Cell Neurosci. 2023;17:1256619. 10.3389/fncel.2023.1256619. 38094513 10.3389/fncel.2023.1256619 PMC10716316

[84] Orvis J, Gottfried B, Kancherla J, Adkins RS, Song Y, Dror AA, et al. gEAR: Gene Expression Analysis Resource portal for community-driven, multi-omic data exploration. Nat Methods. 2021;18:843–4. 10.1038/s41592-021-01200-9. 34172972 10.1038/s41592-021-01200-9 PMC8996439

[85] Wolf FA, Angerer P, Theis FJ. SCANPY: large-scale single-cell gene expression data analysis. Genome Biol. 2018;19:15. 10.1186/s13059-017-1382-0. 29409532 10.1186/s13059-017-1382-0 PMC5802054

