# Supplementary figures and images for: Novel candidate genes for vestibular function identified through GWAS in the hybrid mouse diversity panel
Source: BMC Genomics. 2026 Apr 11;27:479. doi: 10.1186/s12864-026-12810-y (PMC13182000; doi:10.1186/s12864-026-12810-y)

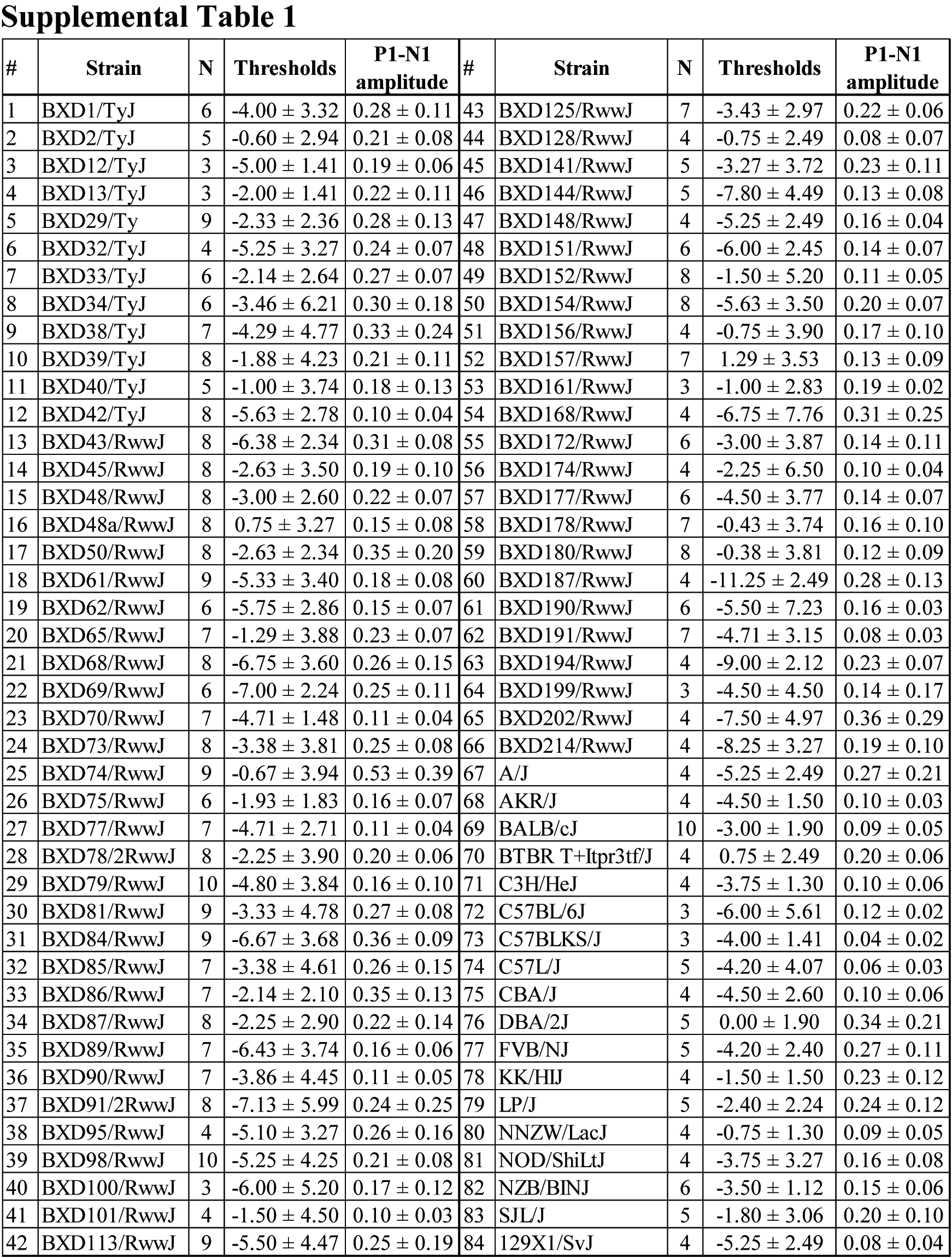

Supplement: Supplementary file 1 — Supplementary Material 1. [file 12864_2026_12810_MOESM1_ESM.tif]

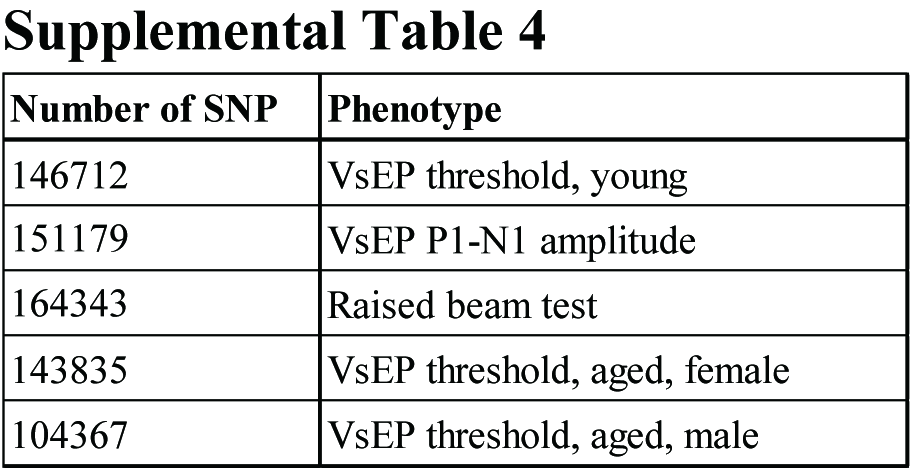

Supplement: Supplementary file 2 — Supplementary Material 2. [file 12864_2026_12810_MOESM2_ESM.tif]

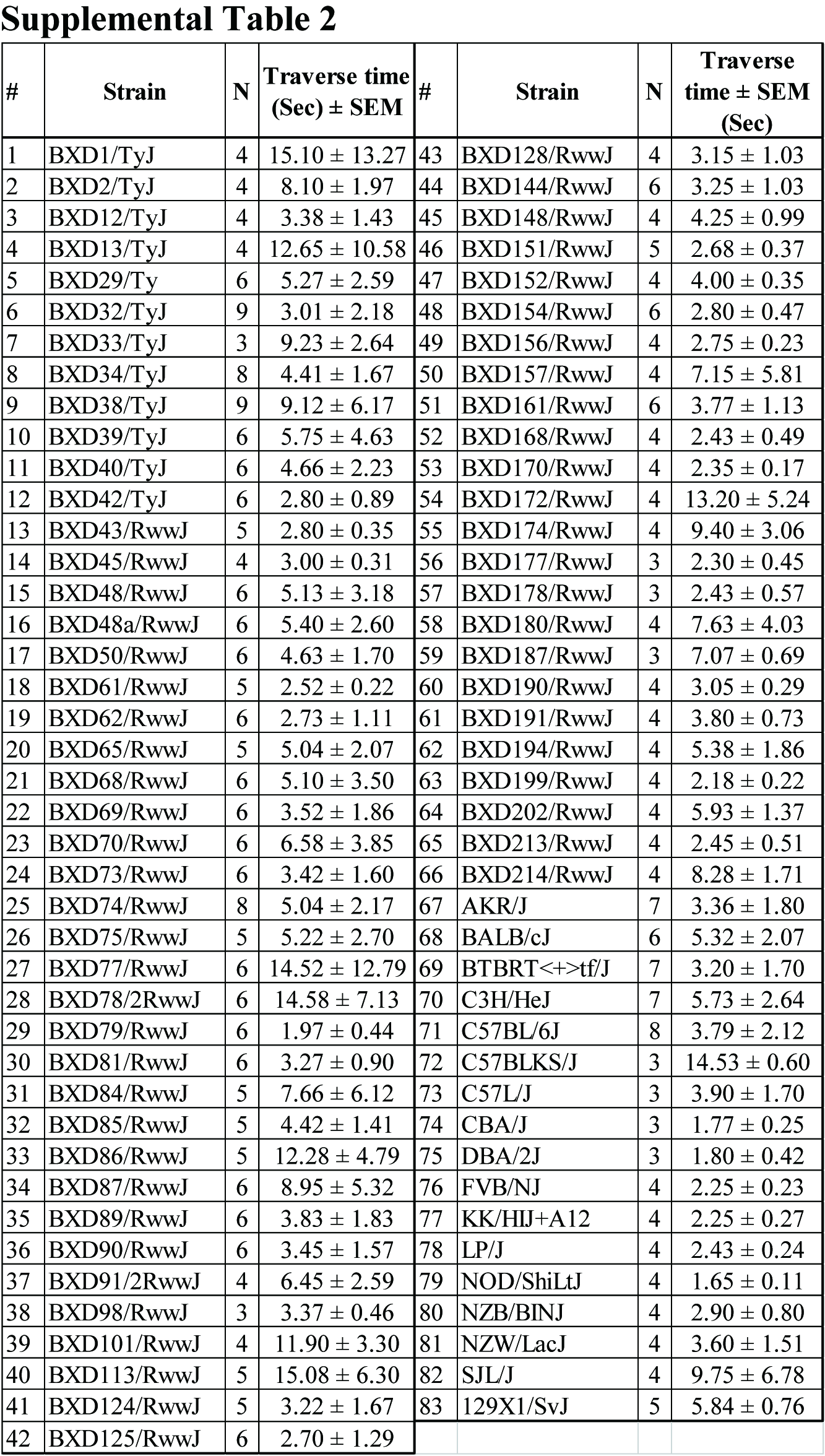

Supplement: Supplementary file 3 — Supplementary Material 3. [file 12864_2026_12810_MOESM3_ESM.tif]

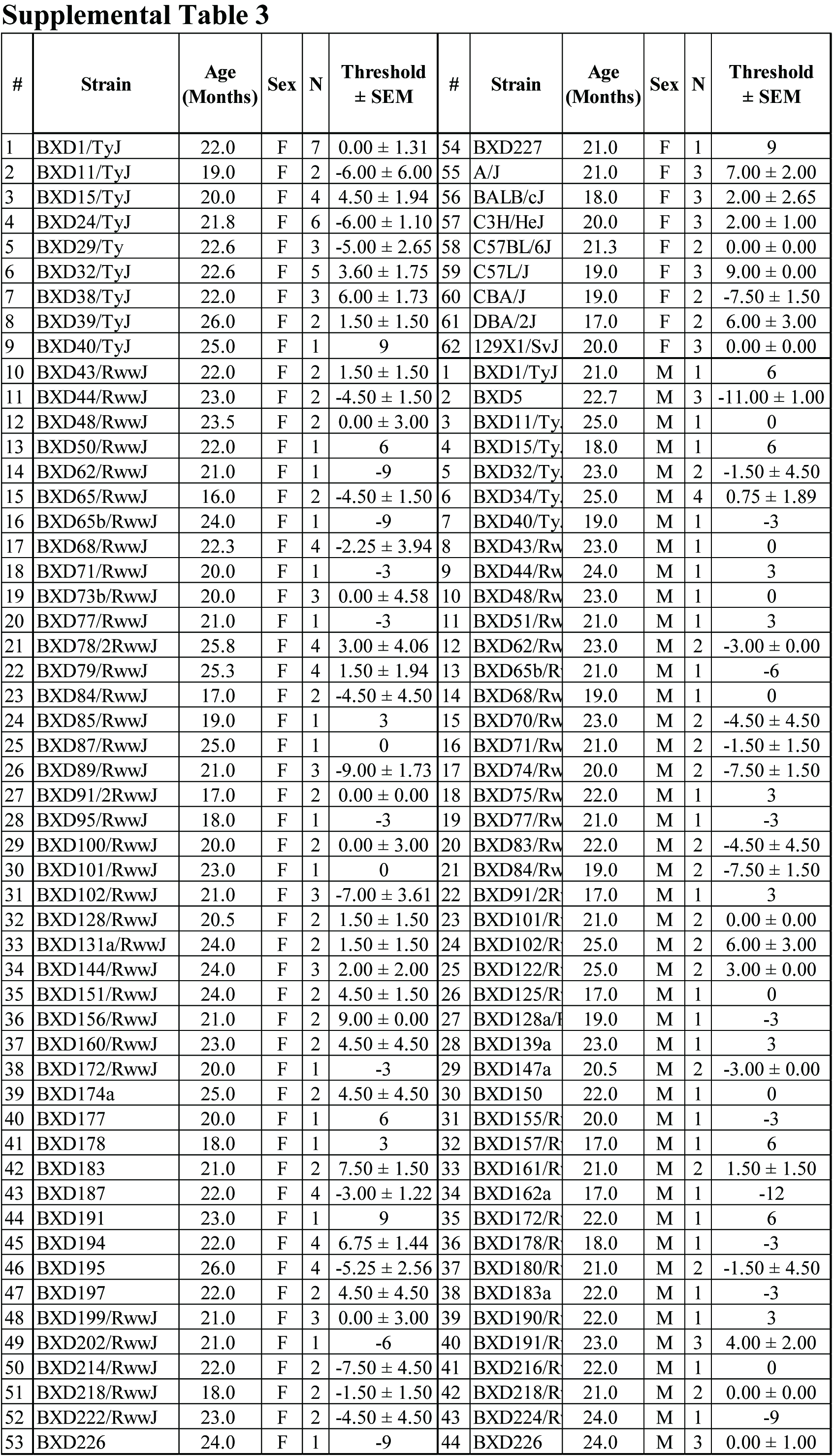

Supplement: Supplementary file 4 — Supplementary Material 4. [file 12864_2026_12810_MOESM4_ESM.tif]

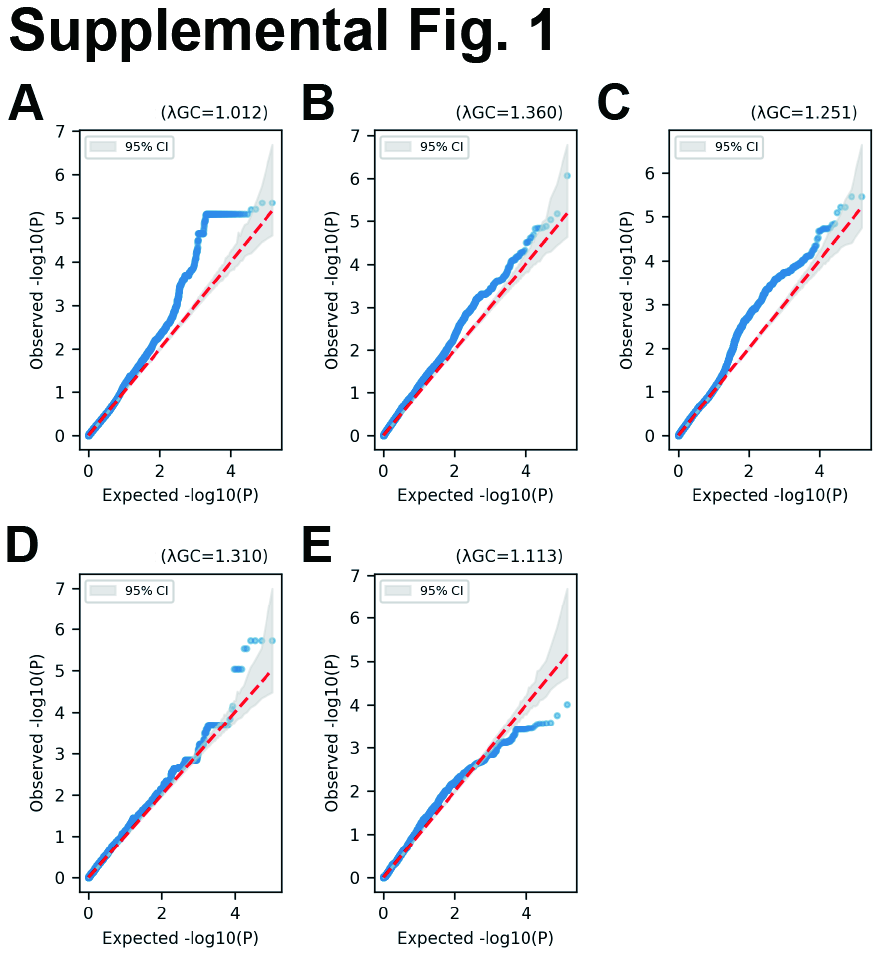

Supplement: Supplementary file 5 — Supplementary Material 5. [file 12864_2026_12810_MOESM5_ESM.tif]

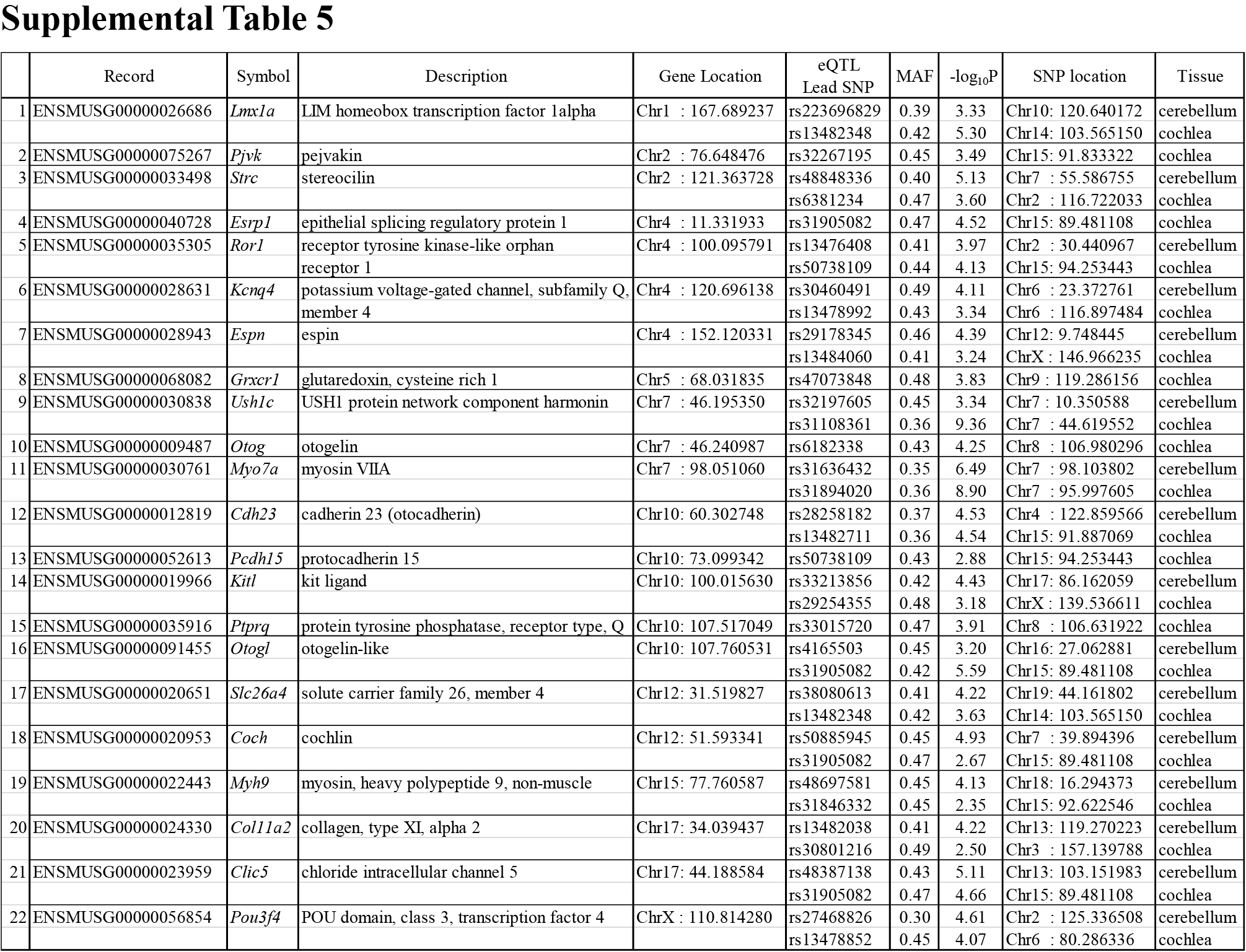

Supplement: Supplementary file 6 — Supplementary Material 6. [file 12864_2026_12810_MOESM6_ESM.tif]
